# Reference tool kinematics-kinetics and tissue surface strain data during fundamental surgical acts

**DOI:** 10.1038/s41597-020-0359-0

**Published:** 2020-01-15

**Authors:** Tyler Schimmoeller, Erica E. Neumann, Tara F. Nagle, Ahmet Erdemir

**Affiliations:** 1 Department of Biomedical Engineering, Cleveland Clinic, Cleveland, Ohio USA; 2 Computational Biomodeling (CoBi) Core, Lerner Research Institute, Cleveland Clinic, Cleveland, OH USA; 3 BioRobotics and Mechanical Testing Core, Medical Device Solutions, Lerner Research Institute, Cleveland Clinic, Cleveland, OH USA

**Keywords:** Musculoskeletal models, Data acquisition, Biomedical engineering

## Abstract

Haptic based surgical simulations are popular training aids in medicine. Previously, surgical tool loads and motion were measured during cutting and needle insertion on non-human tissue and several haptic based simulations were developed to enhance surgical training. However, there was a lack of realistic foundational data regarding the mechanical responses of human tissue and tools during fundamental acts of surgery, i.e., cutting, suturing, retracting, pinching and indenting. This study used four recently developed surgical tools in a variety of procedures on a diverse set of cadaver leg specimens from human donors. The kinematics and kinetics of surgical tools were recorded along with topical three-dimensional strain during commonly performed surgical procedures. Full motion and load signatures of foundational surgical acts can also be used beyond the development of authentic visual and haptic simulations of surgery, i.e., they provide mechanical specifications for the development of autonomous surgical systems.

## Abstract (summary)

**Table:** 

Measurement(s)	kinematics • tissue surface strain • Kinetics • anatomical structure
Technology Type(s)	motion tracking system • digital camera • force transducer • magnetic resonance imaging (MRI) • computed tomography
Factor Type(s)	height • mass • sex • age • experimental condition • race
Sample Characteristic - Organism	Homo sapiens

Machine-accessible metadata file describing the reported data: 10.6084/m9.figshare.11359535

## Background & Summary

Characterizing the interaction between soft tissue and surgical tools is a fundamental part of realistic surgical simulations^1^, accurate robot-assisted surgery^2,3^, and the design of autonomous surgical systems^4^. The resultant force feedback, or haptics, used in simulations and surgeries can be improved with load data from commonly performed surgical techniques. Previous attempts to record realistic tissue-tool interaction during surgical techniques include measuring the forces to cut porcine liver tissue^5,6^ and instrumenting needle insertion^7^, however, the former is limited by its lack of life-like conditions and both limited to a single tool type. More recently, force-sensing bipolar forceps were used to measure forces during brain arteriovenous malformation surgery^8^ and minimally invasive surgical tools were instrumented to provide force feedback in robotic-assisted surgery^9,10^. Haptic based surgical simulations have been developed for a variety of surgical procedures, including endoscopic surgery^11,12^, lumbar puncture^13^, epidural block delivery^14^, laproscopic suturing^15^, cerebral aneurysm clipping^16^ and pre-retinal membrane peel^17^. While haptic feedback is provided during these simulations, there is a lack of quantifiable data used to validate the tissue-tool interaction used within the model. Realistic tissue-tool interaction mechanical data (including tool kinematics and kinetics) would enhance the sense of realism during the mentioned cases and more, with potential to provide a blueprint for devices to automate such surgical procedures^4,18^. Moreover, collecting data on only one or two tools limited to specific use cases would not be sufficient to encompass the many types of surgical procedures.

This study expands the use of previously developed surgical tools (scalpel, forceps, retractor, indenter)^19^ on cadaveric leg specimens varying in donor age, race and body composition to measure realistic loads and motion during eight unique surgical operations. The motion of each surgical tool, in relation to the specimen, creates a complete description of the tool environment, allowing for surgical simulation validation. In addition to load and motion, three-dimensional (3D) tissue surface strain was measured to create a more comprehensive dataset to quantify the mechanical behavior of the tissue during foundational surgical acts.

A related, but separate study using the same cadaveric donors was conducted prior to collection of this dataset^20,21^. The anatomical features of 9 cadaveric arms and legs were captured with computed tomography (CT), magnetic resonance (MR) and ultrasound imaging. Using the instrumented ultrasound, indentation response was captured in each of the anterior and posterior central regions of the upper and lower segments of the specimen (total of 8 sites from each donor). These data may be used in conjunction with the data presented in this study for a more complete description of the anatomical features for each cadaveric leg specimen.

## Methods

### Specimen overview

The right legs of nine cadaver donors (5 males, 4 females) were acquired with the approval of the Human Research Protection Office of the United States Army. Specimen outside the BMI range of 18–25 or with past injuries or surgeries on the right leg were excluded. The height, mass, and demographics were recorded in an experiment configuration file. The average age, height, and weight was 51.6 (39–65) years, 171.6 (145–198.1) centimeters, and 68.0 (45.5–90.7) kilograms, respectively.

### Specimen preparation

**Fig. 1:**
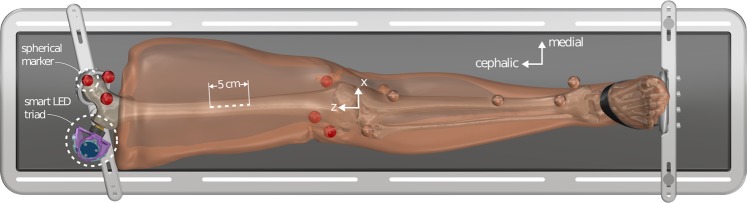
A leg model segmented from a CT scan depicted in the adjustable imaging platform. The spherical markers used in the current study are shown in red on the femur along with motion tracking marker (smart LED triad). Surgical acts were performed on central, anterior region of the upper leg with a target cut length of 5 cm. Femur coordinate system is also shown. The CT scan segmentation was developed from a related study^21^.

Prior to surgical tool experimentation, the specimen underwent CT, MR, and ultrasound imaging as part of a separate but related study^21^. Only the preparation details relevant to the current study will be described. The leg specimens were kept in a −20 °C freezer and removed twenty-four hours before preparation. Once thawed, six 3D printed spheres were implanted using nylon bolts (#8–32) in select locations on the femur (Fig. 1). The specimen was then placed in a custom imaging platform to secure it in a fixed and anatomically neutral position throughout experimentation. After this step the CT, MR, and ultrasound imaging were performed (data available in Schimmoeller *et al*.^20,21^) and the specimen was re-frozen in all but two cases (SMULTIS011 and SMULTIS012), where instead the surgical tool experimentation was performed prior to re-freezing. In all other cases, the specimens were re-frozen and re-thawed at a later date with implanted 3D spherical markers intact.

The specimen was also equipped with smart light emitting diode (LED) motion tracking sensors (Northern Digital Inc., Waterloo, ON). Three clusters of smart LEDs were placed as a rigid triad on the greater trochanter of the femur (Fig. 1).

### Preparation for 3D strain measurement

A high contrast speckle pattern was applied to measure 3D strain during instrumented cutting, pinching, and indenting. The anterior central region of the upper leg was speckled using 0.026′′ sized dots from a stamp supplied by Correlated Solutions (Correlated Solutions Inc., Irmo, SC). The stamp was pressed on the region of interest in three overlapping and but differing orientations so that roughly half of the region was covered with contrasting ink. The ink color (white or black) was selected considering the complexion of the donor skin.

### Experimental setup and data acquisition system

**Fig. 2:**
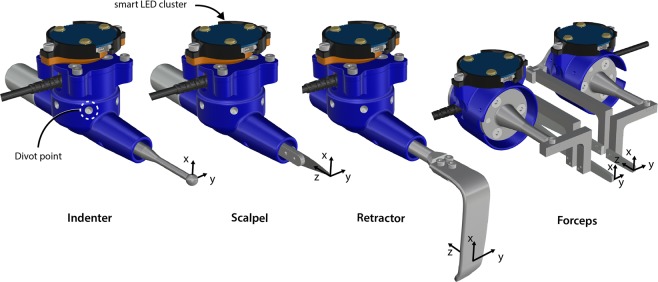
All instrumented surgical tools shown in computer aided design format. The indenter and scalpel shared the same 3D printed handle (blue). The retractor also shares the same 3D handle design however was not used interchangeably. The forceps and retractor tips were cut from the original surgical tool. Spatial load transducers were embedded in the casings allowed recording of tool loads^19^. Motion tracking markers (smart LED triads) and tool coordinate systems are also shown.

The experimental setup consisted of four instrumented tools (Fig. 2), motion tracking system (Optotrak Certus, Northern Digital Inc., Waterloo, ON), main data acquisition computer, VIC-3D computer system with two cameras (Correlated Solutions Inc., Irmo, SC), and data acquisition devices, including a multi-sensor interface box (IFPSMC4 Box, ATI Industrial Automation, Apex, NC) and several multifunction input/output devices (USB-6225, USB-6251, and USB-6289 from National Instruments, Dallas, TX).

Three surgical tools (scalpel, retractor, and forceps) were adapted to accommodate six degree-of-freedom (6-DoF) Nano25 load cells (ATI Industrial Automation, Apex, NC) and smart LED clusters. Additionally, an indenter probe was created to measure bulk tissue response during loading. The three surgical tools were demonstrated in a previous study^19^, as was the custom LabVIEW (National Instruments, Dallas, TX) program^19,22^ for instrumentation of an ultrasound to measure load and track position. The indenter is a 10 mm diameter stainless-steel sphere attached to a stainless-steel cylinder and is interchangeable with the scalpel blade holder and retractor (Fig. 2). The scalpel blade holder and indenter were interchanged as needed. Four load cells and four smart LED clusters were equipped; one for each side of the forceps (each tip measures load and motion independently) and one each for the scalpel and retractor. The load cells were all connected to the multi-sensor interface box (9105-IFPSMC Box, ATI Industrial Automation, Apex, NC) with an SI 250 N calibration (Resolution: F_x_, F_y_ = 1/24 N, F_z_ = 1/8 N, T_x_, T_y_ = 1/660 Nm, T_z_ = 1/1320 Nm). The IFPSMC Box was connected to a data acquisition device (USB-6225, National Instruments, Dallas, TX), which was connected to the data acquisition computer. The motion tracking system, Optotrak Certus (Northern Digital Inc., Waterloo, ON), was connected to the same computer. Around each load cell, there was a 3D printed shield, used to limit the effects of light variation on the load cell output during operation of the tools. The shields and surgical tools had divot points designed into the surface that were digitized to orient the tool relative to its motion tracking smart LED sensor. Raw sensor data from both the load cells (no filtering) and motion tracking smart LEDs were stored, in addition to transformed data. Load data was transformed to each respective tool tip coordinate system (Fig. 2) and motion data was transformed to describe the position of the tool tip origin with respect to the femur coordinate system (defined in Fig. 1).

The VIC-3D system was operated via a separate computer but triggered via the main data acquisition computer by using a 0.5 V electrical pulse, directly triggering the two strain measurement cameras. A second signal was sent from the main data acquisition computer to the VIC-3D computer to temporally synchronize the VIC-3D strain data with the load/motion data. This temporal synchronization signal was received by a data acquisition device (USB-6251, National Instruments, Dallas, TX) and stored in a CSV file containing the time instance of each strain measurement frame and temporal synchronization signal. The signal was also looped back and stored in the load/motion data file created by the LabVIEW software.

### Surgical tool calibration

The surgical tools and VIC-3D system both required calibration prior to experimentation. Each of the tools was spatially related to the leg via the Optotrak motion tracking system. The divot points in each tool were digitized with respect to each tool’s own smart LED cluster. The spherical markers implanted into the femur and bone anatomical landmarks (lateral/medial femoral epicondyles, femoral head [4 points], and lateral/medial tibial plateau) were also digitized to establish a spatial relationship between the femur triad and the anatomical femur coordinate systems. Lastly, the load cells were tared prior to the experiment and any time the tool tips were adjusted or their operation orientation changed, e.g., after scalpel and indenter interchange. Each tool was tared in its 3D printed stand in the approximate orientation of operation: the scalpel/indenter and forceps at 45° (from horizontal) and retractor at 0° (level with horizontal).

The VIC-3D calibration and setup was a multi-step process. First, the cameras were setup approximately 2 feet above the leg aimed at the speckled region so that the center of each camera was aligned to the same dot. The focus and aperture were adjusted accordingly. Next, the spatial relationship between the two cameras was established by capturing roughly twenty images of a calibration plate (supplied by Correlated Solutions) in varying orientations at the same distance from the cameras as the leg. The calibration was then computed by the VIC-3D software.

### Surgical tool operation

The experiment consisted of seventeen operations and eight techniques: indenting, pinching, cutting, widening, closing, everting, suturing and retracting. All operations were performed on the speckled region of the upper leg, and the 3D strain measurement system was only used for the first three trials due to the destructive nature of the third trial (cutting). Details of the operations performed for each trial are included below. The naming of each trial consisted of three parts, each separated by an underscore. See Table 1 for a file naming key. Vernier calipers were used to measure the width, length, and depth of the opening after each trial, starting with the first cut (trial 3). Each subsequent cut trial was along the entire 5 cm original target cut line. All operations were performed by the same researcher.

1. SXX_IND_SKN: The scalpel tool with the indentation insert instead of scalpel blade. The skin was indented roughly 1 cm. First trial of 3D strain measurement (Fig. 3).

**Fig. 3:**
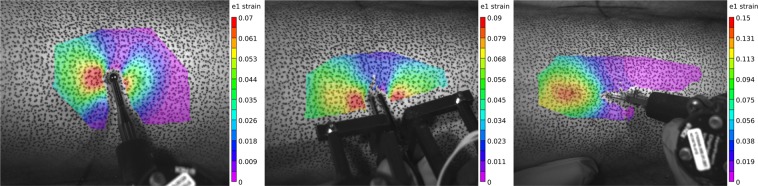
The Lagrangian strain (first principal component) calculated using the VIC-3D software for indenting, pinching, and cutting trials shown left to right, respectively. This data is available as both “.out” and “.csv” file types, as previously mentioned.
2. FXX_PCH_SKN: The forceps tool was used to grasp and lift the skin vertically roughly 1 cm. Collected 3D strain measurement (Fig. 3).
3. SXX_CUT_SKN: The scalpel tool was used with a number fifteen blade to make approximately a 5 cm incision, about 1.5 mm deep in the longitudinal bone direction (proximal to distal) at anterior central location of leg. Last trial of 3D strain measurement (Fig. 3).
4. SXX_CUT_SFI: The scalpel tool cut the skin-fat interface, penetrating the fat only about 1–2 mm.
5. FXX_WID_SFI: The forceps tool was used to grasp the edge of the incised wound and move it from center medially with the left tip towards the center.
6. FXX_CLO_SFI: The forceps tool was used in the same orientation as the previous trial except the incised wound was moved centrally to simulate closing approximation.
7. FXX_EVE_SFI: The forceps tool was used in the same orientation as the previous trial except the skin was everted to simulate eversion prior to suturing.
8. SXX_SUT_SFI: The scalpel tool was used without a blade to close the first knot of a surgeon’s knot (Fig. 4). The suture was first completed up to before the final pulling action to close the knot. The medial side of the suture was grasped by a needle driver and the lateral side to the instrumented scalpel tool at the center of the load sensing axis.

**Fig. 4:**
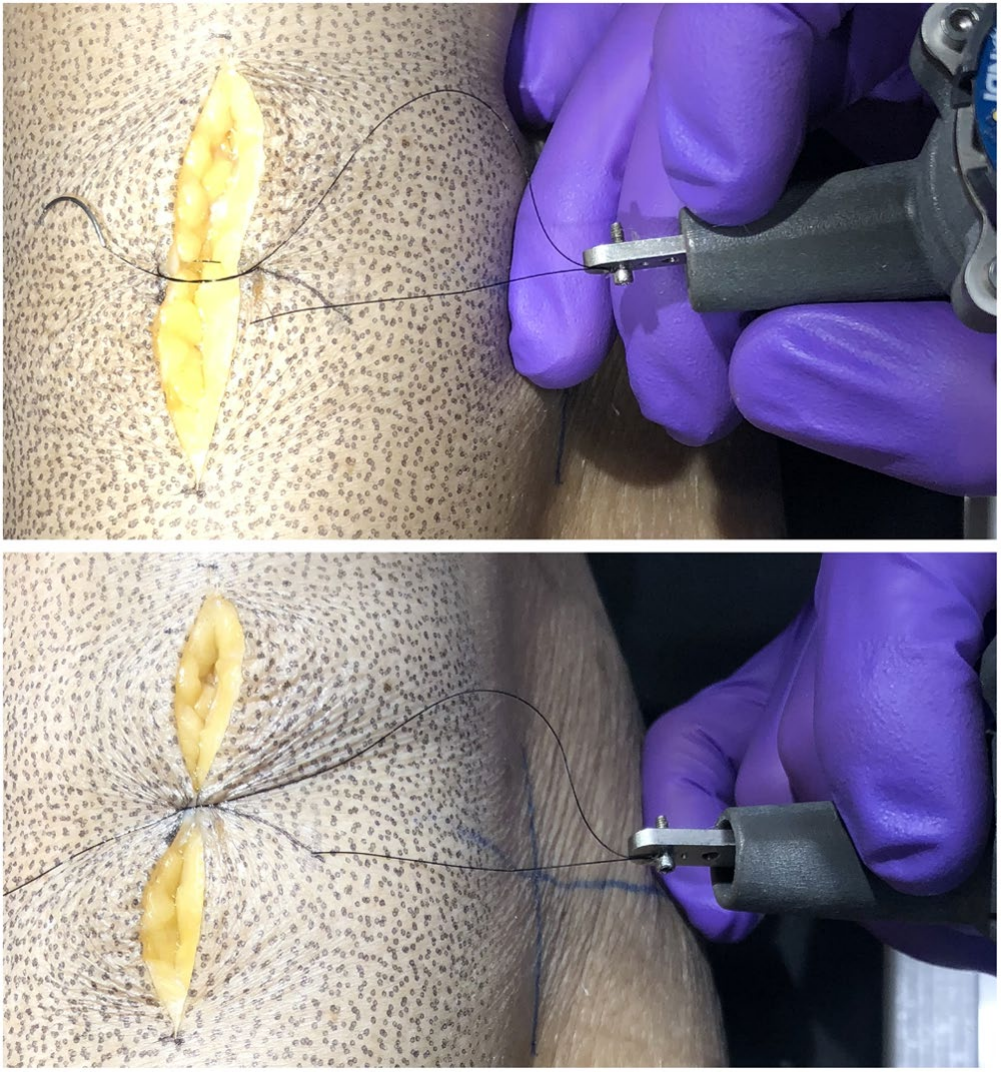
The scalpel blade attachment was used (without blade) to attach the suture directly in line with the tool’s Z-axis. The top image shows the state of the wound and suture prior to closure. The bottom image shows the suture after closer. Note the direction during closure was perpendicular to the axis of the cut (not shown in image).
9. SXX_CUT_FAT: The scalpel tool was used to cut to a depth of approximately 5 mm.
10. RXX_RET_FAT: The retractor tool was used to retract the current full depth of tissue (skin and fat) approximately 2 cm in the medial direction at the center of the incision.
11. SXX_CUT_FMI: The fat-muscle interface was first approached (no data collected) so that for this trial, only the fat-muscle interface was cut and measured.
12. RXX_RET_FMI: The retractor tool was used to retract the tissue at the current full depth (skin, fat, and fat-muscle interface).
13. SXX_CUT_MUS: The scalpel tool was used to cut the muscle to a depth of approximately 5 mm.
14. RXX_RET_MUS: The retractor tool was used retract the tissue at the current full depth (skin, fat, and fat-muscle interface).
15. RXX_RET_MBI: The muscle was first cut (without load measurement) down to the femur. Next, the retractor tool was used to retract the tissue at the current full depth (skin, fat, and muscle).
16. SFX_DIS_FMI: The scalpel and forceps were used to cut along the fat-muscle interface to separate the fat and muscle layers. The forceps grasped the skin while the scalpel was used to cut.
17. SFX_DIS_SFI: The scalpel and forceps were used to cut along the skin-fat interface to separate the skin and fat layers. The forceps grasped the skin while the scalpel was used to cut.

**Table 1:** Explanation of file naming abbreviations.

Tool	Action	Location
SXX - Scalpel	IND = Indent	SKN = Skin
FXX - Forceps	CUT = Cut	SFI = Skin Fat Interface
RXX - Retractor	EVE = Evert	FAT = Fat
SFX - Scalpel/Forceps	WID = Widen	FMI = Fat Muscle Interface
	CLO = Closure	MUS = Muscle
	RET = Retract	MBI = Muscle Bone Interface
	SUT = Suture	
	DIS = Dissect	
	PCH = Pinch	

## Data Records

Imaging, including CT and MRI, that correspond with these data are included with a previous dataset^20,21^. The records for surgical tool kinematics-kinetics and surface strain data^23^ are provided here.

Raw data:

1. *Data –* Tool load and position data in raw form (loads along or about the load cell axes and tool Optotrak sensor positions in the global Optotrak coordinate system) and transformed form (loads in surgical tool tip coordinate system and positions of tool tips in the femur anatomical coordinate system). Data for each tool was stored in each trial’s data file whether it was in use or not (.tdms).
2. *Configuration* – Contains the coordinate system, sensor and state files used during data collection for each data file (.cfg). The coordinate system files define each tool’s unique coordinate system for the software. The sensor and state files provide the structure and description of the binary data file (.tdms) for the raw and transformed data, respectively. Also contains the donor configuration file [.cfg and .xml, which provides the file locations (coordinatesystem.cfg, state.cfg, sensor.cfg, data.tdms) of each data collection site, operation measurements (length, width, depth of incision) and de-identified donor information (demographics)].
3. *VIC3D –* Contains the strain measurement calibration files (.z3d, .tiff) and measurement files (.csv, .z3d, .out, .tiff) for each of the three operations. The project file (.z3d) under calibration provides a pre-processed calibration that may be loaded into an existing VIC-3D project containing the related raw images, or the calibration may be reprocessed using the calibration images. The measurement files contain the electrical signal for temporal synchronization (.csv under AI0), VIC-3D project file (.z3d), raw images from each camera (.tiff), and VIC-3D output data files (.out) plus text document (.csv), containing pixel coordinates, surface displacement, and Lagrange strain tensor data (e_xx_, e_yy_, and e_xy_) for each pixel subset within the area of interest.

**Fig. 5:**
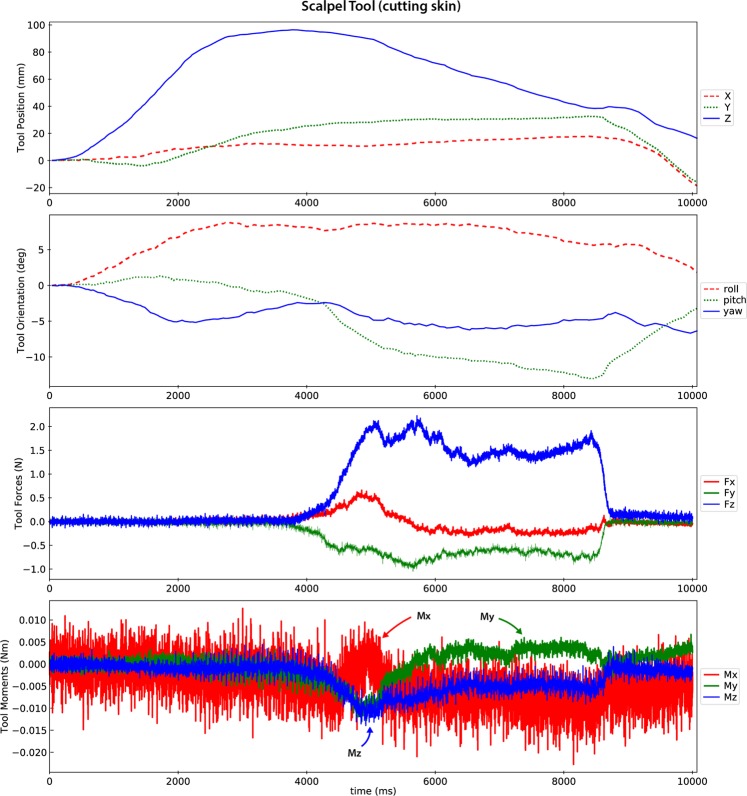
A representative data quality plot showing tool position and orientation in the femur coordinate system and the forces and moments (reaction from tissue onto tool) at the tool tip oriented to the femur coordinate system (refer to coordinate system in Fig. 1). The scalpel approaches the incision site until about 4 seconds, where it is then moved distally to proceed with the cut (008_SMULTIS006-1_SXX_CUT_SKN-2). The load plots show the cutting forces synchronized with the tool motion.

Derivative data:

1. *DataQuality –* Provides a summary plot (Fig. 5) for each trial, contains the load and motion data for the tool used in that trial’s operation. The Optotrak motion is shown in the femur coordinate system (shown in Fig. 1) and the tool load data is shown as the loads at the tool tip origin (shown in Fig. 2), oriented to match the femur coordinate system.

Raw and derivative data files exist for each trial with the following root naming convention: “Experiment Run Number”_”Donor ID””_”Operation Name”-”Trial Number Index”

- Experiment Run Number: 3-digit number that gets auto-incremented for each tdms file (order in which data are collected for each specimen).
- Donor ID: A project name followed by a 3-digit number that gets assigned to each donor in the order that they were acquired. The digit following the donor ID indicates whether the specimen was tested on more than one occasion (always a -1 for this study). The 3-digit donor ID corresponds to the same donor ID of the previous dataset that includes the CT and MR images^21^.
- Operation Name: A set of three abbreviations separated by two underscores. The first indicates the operating tool, the second describes the action, and the third the main tissue of interest (Table 1).
- Trial Number Index: This number indicates the number of attempts for a particular operation. The most recent trial, i.e. highest number, was always used as the final data. Earlier trials were also included in the dataset for completeness.

Additional characters are appended to the root name for further data descriptions. Example file names for a single trial include:

**Table:** 

**Data Acquisition Computer**	
Donor XML	SMULTIS012-1.xml
Sensor configuration	001_SMULTIS004-1_SXX_IND_SKN-1_Sensor.cfg
State configuration	010_SMULTIS012-1_ SXX_IND_SKN-1_State.cfg
Coordinate System	002_SMULTIS004-1_SXX_IND_SKN-1_CoordinateSystem.cfg
Data	001_SMULTIS004-1_SXX_IND_SKN-1.tdms
**VIC-3D Computer**	
Calibration Image	SMULTIS004-cal-0002_0.tif
Calibration Project File	SMULTIS004-cal.z3d
Calibration Analog Data	SMULTIS004.csv
Surgical Act Raw Image	SXX_CUT_SKN-0029_1.tif
VIC-3D Data File	SXX_CUT_SKN-0029_0.out
Derivative Data Export File	SXX_CUT_SKN-0029_0.csv
Surgical Act Analog Data	SXX_CUT_SKN.csv
Operation Project File	SXX_CUT_SKN.z3d

## Technical Validation

The load measurement and motion capture systems were integrated to provide synchronous load and motion data. Their respective sampling rates were 1000 Hz and roughly 30 Hz. A tap test was performed to check temporal alignment by tapping each tool with enough force to abruptly move it. Ideally, the load cell and motion sensor would record the movement via load and motion at the same time. Two taps were analyzed on each tool configuration by locating corresponding peaks using a custom Python script. The mean and standard deviation for the indenter, scalpel, forceps and retractor were 71.5 +/− 32.5 ms, 49.5 +/− 19.5 ms, 48.5 +/− 22.5 ms, and 32.5 +/− 23.5 ms, respectively. The time difference was comparable to the lowest measurement frequency and assumed to be adequate for data analysis without correction. Additionally, each unique surgical operation was simulated in-air under zero-load to show a baseline load and movement profile. The tools were tared in their stands prior to each trial just to approximate their orientation as they would be for real experimentation, therefore a change in orientation, such as the rotation of the forceps during eversion, the measured load would change due to the change in center of gravity of the tool. The tap test and zero-load test data, scripts, and results can be found in the source code repository (https://simtk.org/svn/multis/studies/SurgicalToolsRefData/).

Load cell drift was inherently captured throughout testing, by the inactive tools. We examined the raw load cell signal of the left forceps, which was inactive, across the later 10 trials of SMULTIS004-1. The average load and torque for each channel was calculated and used to examine the drift. The range (difference between the minimum and maximum) of the average loads were 0.016 N, 0.096 N, and 0.447 N for the x, y, and z directions, respectively. The range of the average torques were 0.844E-3 Nm, 0.246E-3 Nm, and 0.231E-3 Nm, for the x, y, and z axes, respectively. The drift from trial to trial can also be eliminated by applying a digital tare for each 20 second trial, using the initial 0.5 seconds of data, when the tool is not in contact with the tissue.

## Usage Notes

All raw and derivative data described above can be found in a dynamic data management and querying site (https://multisdelta.stanford.edu/). A static version of the data is also provided^23^.

More detailed specifications regarding Python scripts, hardware integration and design, the LabVIEW program and more can be found on the project website (https://simtk.org/plugins/moinmoin/multis/). A source code repository with downloadable scripts and data can be found at (https://simtk.org/svn/multis/).

## Data Availability

A source code repository is available at (https://simtk.org/projects/multis), which provides scripts to parse the data files, create data overview plots, and register surgical tools data to the image (MRI or CT) coordinate systems. Install Python 2.7 with Anaconda (https://docs.anaconda.com/anaconda/) and the npTDMS library (reading LabVIEW data files). Additional libraries can be installed using Anaconda (“conda install” command). A static download package is also available containing all raw and derivative data pertinent to the current study^23^.
